# Medical Cannabis Use and Pain: An Experience Sampling Study

**DOI:** 10.3389/fpsyt.2021.728283

**Published:** 2021-10-27

**Authors:** Sharon Rodner Sznitman, Dennis Rosenberg, Simon Vulfsons, David Meiri, Talya Greene

**Affiliations:** ^1^School of Public Health, University of Haifa, Haifa, Israel; ^2^Institute for Pain Medicine, Rambam Health Care, Haifa, Israel; ^3^Rappaport School of Medicine, Technion Israel Institute of Technology, Haifa, Israel; ^4^Department of Biology, Technion Israel Institute of Technology, Haifa, Israel; ^5^Department of Community Mental Health, University of Haifa, Haifa, Israel

**Keywords:** chronic pain, experience sampling methods, medical cannabis, pain, intention to use, THC - tetrahydrocannabinol

## Abstract

**Background:** Little research has tested associations of pain and MC use after long-term treatment and through methods that have external validity outside experimental settings. The study examined associations of pain, associated painful experiences, and long-term medical cannabis (MC) use in chronic pain (CP) patients using a naturalistic daily diary study that provided novel and externally valid data.

**Methods:** Data were obtained from 78 MC users with CP three times daily over a 10-day period (n_observations_ = 1,688). Mixed-effects models were used to test the associations between MC use and momentary experiences of pain, affect, and fatigue.

**Results:** Within persons, elevated experiences of pain intensity were associated with greater intention to use MC within the next hour. No evidence was found that the time lapse since last MC use was associated with pain levels, negative affect, or fatigue.

**Conclusions:** The results imply that after long-term use, CP patients intend to use MC in response to pain experiences. Yet, they may not actually achieve the pain relief. More research is needed to examine whether continued MC use despite lack of pain relief is related to relief of other symptoms (e.g., dependence, withdrawal) or positive benefits (e.g., general sense of well-being) or tolerance.

## Key Points

**Question:** Are elevated pain experiences related to higher intention to use medical cannabis, and are pain experiences lower immediately after medical cannabis use compared to when longer time has passed since last medical cannabis use?

**Findings:** Within persons, elevated experiences of pain intensity were associated with greater intention to use medical cannabis within the next hour, but no evidence was found that the time lapse since last medical cannabis use was associated with pain levels.

**Meaning:** Chronic pain patients who have used medical cannabis for extended periods intend to use medical cannabis in response to pain experiences but pain relief may not be achieved.

## Introduction

The prevalence and refractory nature of chronic pain (CP) in the population, and growing disfavor of opiate-based treatments (1), have generated interest in alternative therapies, including medical cannabis (MC). Cannabinoids have been shown to have some analgesic effect, but also to give significant side effects (2, 3). CP is associated with co-morbidities, some of which have been examined as secondary outcomes in RCTs testing MC analgesic effects. Moderately strong evidence shows that orally administrated synthetic cannabinoids improve short-term sleep outcomes (4). Some studies report significant improvement in anxiety and depression (5) through cannabinoids compared to placebos, while others report little or no effect (6). Cannabinoids have been found to have little, mostly non-significant, effect on quality of life (7, 8).

The external validity of RCTs testing MC analgesic effect remains debatable, with most study periods limited to <20 weeks (9), measuring the effect of only one or two cannabinoids, i.e., Δ9-tetrahydrocannabinol (THC) and cannabidiol (CBD), with patients typically instructed to use pre-set MC doses and administration modes. These conditions bear little resemblance to real-life MC use among CP patients. MC for CP is typically used long-term, and usually administered *ad-libitum*, and as needed. MC is commonly administered by smoking whole plant products, not ingesting cannabinoid extracts. Real-life MC use factors may bear on MC effects on pain and associated co-morbidities, suggesting that studies which examine short-term use of non-inhaled THC/CBD-extracts are not optimal.

While RCT literature reports slight analgesic effect, CP patients report considerable benefit from MC (9). Intention to use MC and perceived benefit therefrom may arise from other domains, such as improvement in fatigue or mood. Yet, the literature does not support this either. Given growing use of MC among CP patients, the association between pain and MC use was examined through research designs with strong external validity. An experience sampling method (ESM) study was conducted to examine relations between intention to use MC within the next hour, time passed since last use, and momentary pain experiences, negative affect and fatigue in the everyday lives of Israeli CP patients authorized to use MC products of their choice *ad libitum*.

ESM is a data capture technique involving repeated sampling across multiple days of momentary experiences (e.g., pain, MC use) as close in time to the experience as possible in a naturalistic environment. ESM reduces recall bias and facilitates accurate observations of pain and related experiences, dosage, and timing of MC use in real-life contexts (10). The observational nature of ESM allows study of patient-directed MC use, rather than research-mandated protocols. Intensive within-person ESM assessments can illuminate the dynamics of fluctuating symptoms and MC use (10). ESM enables analyses of covariations between MC use, pain, mood and fatigue experiences estimated in the data rather than involving retrospective judgments. Since acute effects of MC are relatively short-lived, ESM offers the granularity necessary to detect associations possibly lost in global retrospective reports.

Only one study, which has used ESM to capture pain and cannabis use, was found (11). It involved 182 non-licensed recreational cannabis users for whom elevated pain experiences were not associated with cannabis use occasions at between- or within-person levels. However, once participants were using cannabis, they consumed larger amounts when experiencing elevated pain. Some studies have used ESM to examine associations between pain and prescription opioids use. One found that elevated pain experiences were associated with increased likelihood of opioids use in lower-back CP patients, with participants reporting reduced pain following use (12). A study with college students found that higher momentary pain was positively associated with intentions to engage in prescription opioids misuse (13).

Building on the literature, it was hypothesized that when patients experience higher pain levels, negative affect and fatigue, they would be more likely to report intention to use MC within the hour compared to when they experienced less pain, negative affect and fatigue. It is also expected that when patients report shorter time-lapse since last use, they would report lower pain severity, negative affect and fatigue compared to when they report longer time-lapse.

Beyond testing these main effect hypotheses, moderation by sex and MC administration mode was also tested. Preclinical evidence suggests that cannabis analgesic effect may be greater in women, but published RCTs examine cannabis effect either in one sex, or did not include sex in data analyses (14). Exceptions include one RCT examining analgesic effects of Nabilone (synthetic cannabinoid mimicking THC) (15), which found that treatment relieved hyperalgesia responses for women, but not men. Another RCT found the opposite, with men, but not women, exhibiting an analgesic response to capsule-administered THC to a non-clinical sample of cannabis users (16). A naturalistic observational study found greater perceived MC analgesic efficacy for headaches and migraines in women over men (9). Therefore, exploratory models to study sex differences were examined.

Inhaled administration is rapidly absorbed into the bloodstream (peak effects within minutes, with rapidly declining effects over 30 min and a plateau lasting two to 4 h), while other routes are slower and longer lasting (17). Therefore, exploratory models testing associations between exclusive and non-exclusive administration by inhalation were examined.

## Methods

### Sample and Procedures

Over half of Israel Ministry of Health MC licenses are issued for a CP indication (18), including “complex regional pain syndrome (CRPS),” neuropathic pain, and fibromyalgia (19). Using the MC database of the Israel Institute of Technology Cancer Biology and Cannabinoid Research Laboratory, licensed individuals aged 18 or older with smartphones who used inhalation as their main MC administration method for a CP condition, were recruited. Eligible participants who responded to an initial email survey were contacted by a trained research assistant who explained the baseline assessment and ESM, obtained verbal consent, and transmitted a Qualtrics link to the baseline survey (about 15 min). Starting 1 week later, participants, who completed the baseline survey, received a short (1–2 min) survey link, three times daily (8 A.M., 2 P.M., and 8 P.M.) for 10 consecutive days. Each short survey, with a link valid for 3 h, measured momentary pain, related symptoms, timing of last MC use, and intention to use MC in the next hour. Participants were reimbursed with a gift voucher (100 NIS, ~30 USD). The study protocol was approved by the Ethics Committee of the Faculty of Welfare and Health Sciences at the University of Haifa (certificate #304/19). The manuscript adheres to the applicable CONSORT guidelines.

**Figure 1:**
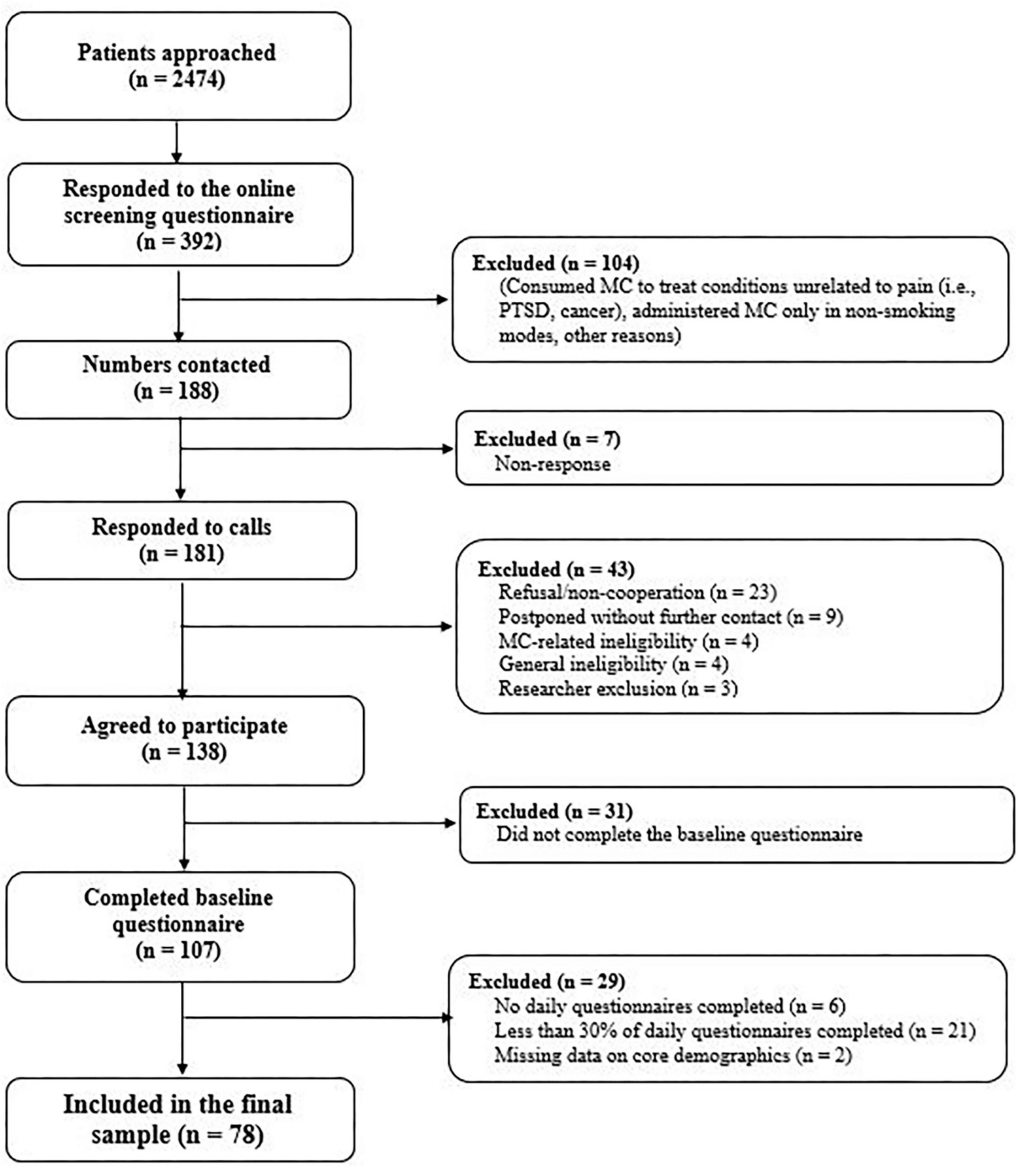
Flow of patient through the study.

Sample recruitment flow is presented in Figure 1. The analytical sample of 78 CP patients was derived from 107 respondents. Those completing <30% of daily surveys (*n* = 27) were excluded (unreliable data) (20). Two were excluded due to missing socio-demographic data. Participants completed a mean of 21.6 assessments (min = 9, max = 30; SD = 5.7). Non-response, more prevalent later on in study (response fatigue), was unrelated to demographic variables (age, gender), baseline pain interference, or MC use patterns (length of use, administration mode, quantity, frequency).

### Measures

Baseline demographics and health parameters included sex, age, prescribed opiate use (0 = No, 1 = Yes), length of MC use (in months), typical frequency of MC use, whether MC was administrated exclusively through inhalation (smoking/vaping). Participants were asked to identify which of 24 physician-designated diagnoses and pain symptoms were applicable. Respondents were also asked to estimate how much MC had helped to reduce their pain in the last week and the extent to which their pain had interfered with their work performance (including the outdoor work and home errands) during the last month (0 = not at all-−5 = to a great extent.

Momentary assessments of MC use: Intention to use MC (“Do you intend to use MC in the next hour?,” 0 = No, 1 = Yes) and time passed since last use (“How much time has passed since you last used cannabis,” ranging from “0” = <1 h, to “6” = 6 h or more).

Momentary assessments of pain: (a) *Pain intensity* (“How much pain are you feeling at the moment?”—from “0” = no pain at all, to “10” = the strongest pain imaginable); (b) *Pain interference* (“How much does your pain interfere with what you are doing at the moment?”—from “0” = does not interfere at all, to “10” = interferes to the highest level possible). “0” responses were coded as missing information.

Momentary assessments of negative affect and fatigue were measured by selected items from the PANAS-X Positive and Negative Affect Schedule (21). Negative affect was measured by asking participants how much they felt the following at the moment: nervous, lonely, sad, guilty, anxious and under pressure (from “1” = not at all, to “4” = a great deal). The means of the items were stored and log transformed after ladder of powers testing indicated that the transformation approximated the normal variable distribution more closely and minimized risk of Type II error. Reliability was high (Cronbach's alpha = 0.9). Consistent with previous research (22), fatigue was considered independent of negative affect, and was measured with a single item (“How tired are you feeling at the moment?” from “1” = not at all, to “4” = a great deal).

### Data Analytic Plan

Means, standard deviations and proportions were obtained using SPSS 25.0 (23). Multivariate analyses were conducted in Stata (24) using mixed-effects models that distinguished within-person variations from between-person variations. Between-person versions of each momentary independent variable were created by calculating the study period mean for each participant. Within-person versions of these variables were created by subtracting person mean variables from raw scores. Between and within versions of independent variables were entered into the models.

In the first set of analyses, a logistic mixed-effects model was run predicting intention to use MC, with the within- and between-person versions of pain intensity, affect, and fatigue variables as predictors. Pain interference was excluded to avoid collinearity problems as it was correlated with pain intensity (*r* = 0.79). “Hours passed since last MC use” may be associated with both independent variables and intention to use cannabis (dependent variable). Accordingly, between and within-person versions of this variable were controlled.

In the second set of analyses, four linear mixed-effects models were run to understand momentary pain intensity, pain interference, negative affect, and fatigue. The independent variable of interest was time-lapse since last MC use, both between-person and within-person. In the main models, this variable was entered as a linear variable. To capture varying associations between time-lapse since last use and dependent variables, the squared form of the variable (U-shaped time association) and categorical cut-offs: use <1, 2 or 3 h ago vs. more were tested.

All models included covariates: sex, age, opiate use, exclusive inhalation administration, MC treatment length, time of day (morning [referent], afternoon, evening), and linear survey time effects measured as an ordinal variable ranging from 1–30 per prompt. Exploratory analyses included cross-level interactions for time-lapse since last use, and between-person variables: sex, and exclusive inhalation administration. Random intercepts and slopes models were estimated using a first-order autoregressive covariance structure to account for autocorrelation in repeated measures. Missing values were handled by listwise deletion. Marginal R (2) was calculated to estimate explained variance (25). To measure local effect sizes (26) the difference was calculated in within-person intercept variance between models with and without momentary pain experience predictors (model predicting intention to use MC), and with and without time passed since last use (models predicting pain-related experiences), but with all other covariates. The difference was divided by the within person intercept variance of the models without the momentary pain experiences/time since last use.

## Results

### Sample Description

Participants' average age was 43.3 years (SD = 10.6); 56.4% were male. A quarter of participants reported prescription opiate use in the month prior to participation, and 57.7% reported exclusive inhalation administration. Patients had been authorized MC use for 3.7 years on average (3–124 months). Diagnoses and pain symptoms were chronic low-back pain (CLBP) (43.6%), complex regional pain syndrome (CRPS) (30.8%), paresthesia (29.5%), fibromyalgia (28.2%), chronic fatigue (21.8%) (Table 1). The majority (72.2%) reported having more than one diagnosis. Patient's average estimated pain reduction due to MC use during the last week was close to 4 (mean = 3.80, S.D. = 0.20), representing an estimated moderate pain reduction. Furthermore, the sample average estimated pain interference last month was 3.5, representing the midpoint between “moderate” and “large” pain interference.

**Table 1:** Sample characteristics.

**Parameters**	**N**	**Values**
**Socio-demographics**		
Male sex, *n (%)*	78	44 (56.4)
Age, mean (S.D.)	78	43.3 (10.6)
**Pain related parameters**		
Chronic lower back pain (CLBP), *n (%)*	78	34 (43.6)
Complex regional pain syndrome (CRPS), *n (%)*	78	24 (30.8)
Paraesthesia, *n (%)*	78	23 (29.5)
Fibromyalgia, *n (%)*	78	22 (28.2)
Chronic fatigue, *n (%)*	78	17 (21.8)
Prescription opiate use, *n (%)*	78	20 (25.6)
Estimated last week pain reduction from MC use, mean (S.D)	78	3.8 (0.20)
Last month pain interference with work performance, mean (S.D.)	78	3.5 (1.1)
**MC use**		
Exclusive MC administration by inhalation, *n (%)*	78	45 (57.7)
Drops, *n (%)*	78	25 (32.1)
Eating or drinking, *n (%)*	78	12 (15.4)
Concentrates, sublingual spray or topical, *n (%)*	78	2 (2.6)
Months with MC license, mean (S.D.)	78	43.9 (32.4)
No. of cannabis cigarettes smoked on average per day, mean (S.D.)	78	4.3 (3.1)
Daily MC use, *n (%)*	78	69 (88.5)
**Momentary attributes**		
Proportion of times participants intended to use MC in next hour	1,684	953 (56.7)
Average hours lapsed since last MC use, mean (S.D.)	1,682	2.6 (2.5)
Average pain intensity, mean (S.D.)	1,687	4.5 (2.6)
Average pain interference, mean (S.D.)	1,552	3.8 (2.8)
Average negative affect, mean (S.D.)	1,688	1.5 (0.6)
Average fatigue, mean (S.D.)	1,688	2.3 (1.0)

*MC, Medical Cannabis; S.D., Standard Deviation; 4, The following momentary missing patterns were observed: intention to use cannabis in next hour; 6, Hours lapsed since last MC use; 1, Pain intensity; 3, Pain interference*.

### Descriptive Summary of Momentary Data

Participants reported intention to use MC within the next hour 57% of the times prompted. On average during the study period, respondents reported that 2.6 h had passed since last use (range 0–6), mean momentary pain intensity of 4.5 (range 0–10), mean pain interference of 3.8 (range 0–10), mean negative affect of 1.5 (range 1–4), mean fatigue of 2.3 (range 1–4).

### Momentary Associations: Intention to Use MC

The model predicting intention to use MC within the hour explained 25% of the variance in intention, yet the local effect size for within-person variables of interest (momentary pain experiences/negative affect/fatigue) was small = 0.04 (Table 2). Intentions were higher in the afternoon and evening compared to morning, and became lower as the study progressed. Mean time-lapse since last use, and mean differences in pain experiences (intensity/fatigue/negative affect) were not associated with intention to use. However, at time points when participants reported higher pain intensity they were more likely to report intentions to use MC within the hour compared with when they reported lower pain intensity. When participants reported higher negative affect, they were less likely to report intentions to use within the hour compared with when they reported lower negative affect. Within-person fluctuation in fatigue was not related to intentions to use.

**Table 2:** Results from mixed effects models predicting intention to use cannabis in the next hour (*N* = 78).

	**Estimate (SE)**	***z***	***p*-value**
Intercept	0.275 (1.224)	0.220	0.823
Between participants pain intensity	0.009 (0.103)	0.090	0.928
Within participants pain intensity	**0.340 (0.048)**	**7.080**	** <0.001**
Between participants fatigue	−0.184 (0.391)	−0.470	0.639
Within participants fatigue	0.049 (0.084)	0.580	0.559
Between participants negative affect	1.224 (0.672)	1.820	0.069
Within participants negative affect	**−1.010 (0.367)**	**−2.750**	**0.006**
Between participants hours since last cannabis use	−0.194 (0.129)	−1.510	0.132
Within participants hours since last cannabis use	0.023 (0.029)	0.780	0.433
Male	0.197 (0.402)	0.490	0.625
Age	−0.002 (0.017)	−0.150	0.883
Opiate use	−0.080 (0.210)	−0.380	0.704
Exclusive administration of cannabis through inhalation	0.112 (0.391)	0.290	0.775
Months with MC license	0.006 (0.006)	0.900	0.367
Afternoon (morning = reference)	**0.523 (0.145)**	**3.590**	** <0.001**
Evening (morning = reference)	**1.551 (0.160)**	**9.690**	** <0.001**
Linear time trend	**−0.017 (0.009)**	**−1.990**	**0.047**
Marginal R^2^	0.250		

*MC, Medical Cannabis; SE, Standard Error*.

*Bold values indicates significant results*.

### Momentary Associations of Hours Elapsed Since Last MC Use and Symptoms

Overall, the models predicting pain, affect and fatigue outcomes explained between 3 and 12% of the variance in outcome variables. The local effect sizes for within-person variables of interest were minimal (pain intensity = 0.00; pain interference = 0.01; negative affect = 0.00; fatigue = 0.00) (Table 3). The variable indicating within-person differences in time-lapse since last use was not associated with pain, affect or fatigue outcomes. Participants who on average reported greater time-lapse since last use reported more negative affect. Pain and fatigue outcomes were not associated with between-person differences in time-lapse since last use.

**Table 3:** Results from mixed effects models predicting pain experiences, negative affect and fatigue.

	**Model 1**			**Model 2**			**Model 3**			**Model 4**		
	**Pain intensity**			**Pain interference**			**Negative affect**			**Fatigue**		
	**Estimate (SE)**	***z***	***P*-value**	**Estimate (SE)**	***z***	***P*-value**	**Estimate (SE)**	***z***	***P*-value**	**Estimate (SE)**	***z***	***P*-value**
Intercept	5.260 (1.241)	4.24	<0.001	4.376 (1.245)	3.51	<0.001	0.126 (0.170)	0.74	0.459	2.545 (0.314)	8.11	<0.001
Between participants hours since MC use	0.223 (0.180)	1.24	0.216	0.232 (0.183)	1.27	0.204	**0.051 (0.025)**	**2.07**	**0.038**	0.062 (0.045)	1.37	0.172
Within participants hours since MC use	−0.021 (0.016)	−1.35	0.177	−0.016 (0.022)	−0.74	0.457	0.002 (0.002)	1.05	0.293	0.006 (0.009)	0.68	0.500
Male	**−1.057 (0.513)**	**−2.06**	**0.040**	−0.499 (0.514)	−0.97	0.332	0.037 (0.070)	0.53	0.596	**−0.496 (0.129)**	**−3.84**	** <0.001**
Age	−0.016 (0.024)	−0.64	0.523	−0.005 (0.025)	−0.22	0.826	0.001 (0.003)	0.33	0.743	−0.002 (0.006)	−0.36	0.718
Opiate use	0.075 (0.124)	0.6	0.548	0.150 (0.176)	0.86	0.393	0.012 (0.016)	0.77	0.44	−0.041 (0.066)	−0.63	0.528
Exclusive administration of cannabis through inhalation	−0.682 (0.539)	−1.27	0.206	−0.147 (0.541)	−0.27	0.785	−0.068 (0.074)	−0.92	0.355	**−0.324 (0.136)**	**−2.39**	**0.017**
Months with MC license	0.010 (0.009)	1.15	0.25	0.006 (0.009)	0.62	0.534	0.001 (0.001)	0.44	0.657	0.003 (0.002)	1.42	0.157
Afternoon (morning = reference)	0.081 (0.076)	1.07	0.286	**0.285 (0.110)**	**2.6**	**0.009**	0.000 (0.10)	0	0.996	0.072 (0.044)	1.65	0.100
Evening (morning = reference)	**0.325 (0.078)**	**4.16**	** <0.001**	0.167 (0.113)	1.48	0.138	−0.009 (0.010)	−0.88	0.376	**0.163 (0.045)**	**3.63**	** <0.001**
Linear time trend	**−0.026 (0.005)**	**−4.97**	** <0.001**	**−0.014 (0.007)**	**−1.99**	**0.046**	0.000 (0.001)	0.3	0.762	**−0.008 (0.003)**	**−2.99**	**0.003**
*N*		78				78		78			78	
Marginal R^2^		0.09				0.03		0.05			0.12	

*MC, Medical Cannabis; SE, Standard Error*.

*Bold values indicates significant results*.

Men reported less pain intensity and fatigue than women. Exclusive inhalation administration was associated with less fatigue. Participants were more likely to report pain interference in the afternoon compared to in the morning, and in the evening respondents reported more pain intensity and more fatigue than in the morning. Participants reported less pain intensity, pain interferences, and fatigue but not less negative affect, as the study progressed.

The main model's results were consistent with analyses that tested associations of a U-shaped variable of time-lapse since last use as well as that occurring <1, 2, and 3 h, concerning each outcome. No within-person versions of these “time since last use” variables were associated with pain, affect or fatigue. Sex and exclusive inhalation administration were not moderators for any outcomes.

## Discussion

Results show that when long-term MC users experience high pain intensity levels, they are more likely to report intention to use MC within the next hour compared to when pain levels are lower. When participants experience greater negative affect, they are less likely to report intention to use MC within the hour compared to when negative affect is lower. Within-person fatigue variations were not associated with intention to use MC. These results suggest that patients use MC in a functional manner and as prescribed, namely to medicate pain symptoms as opposed to other comorbid symptoms, such as coping with momentary experiences of elevated negative affect or fatigue, although the effect size for within-person variables was small.

While it may seem surprising that more negative affect related to less intention to use MC, it needs to be noted that this association was found net of (e.g., while controlling for) within-person fluctuations in pain intensity. Thus the finding suggests that higher negative affect, that is unrelated to pain, relates to lower intention to use MC. It is possible that this type of momentary negative affect is associated with contexts that make MC use unfeasible, for instance during work hours or while attending to small children or driving a vehicle which lowers intention to use MC. We do not have data on the specific situation that respondents were in when reporting on momentary experiences and thus we cannot explore this further. More research in this area that includes data on situational context is warranted.

The analyses show that, net of pain experiences, intention to use MC within the hour was higher in the afternoon and evening compared to morning. As cannabis is used as a sleep aid (27), and sleep problems are a common CP comorbidity (28), higher evening intention to use MC may relate to sleep issues. Higher afternoon and evening intention to use MC, net of pain experiences, may also relate to convenience, while work commitments, child care, etc., may deter morning use.

Surprisingly, time-lapse since last use was not associated with momentary pain, affect or fatigue across the overall sample. Given research on digestive time and other time-related subjective effects of inhaled cannabis (29), various cut-off points and linear and cubic associations of time-lapse since last use were tested, but no evidence that time elements are associated with momentary pain symptoms, negative affect or fatigue was found. Interactions with a between-subject indicator for exclusive inhalation administration showed no significant association with momentary symptoms.

This suggests that some patients use MC without a clear pain association or other symptom reduction, further suggesting that other benefits, not measured in the current study, may be achieved, e.g., improved sleep, increased positive affect, sense of well-being. The literature suggests that MC may reduce pain at the outset of treatment, but tolerance may develop after long-term use (30, 31), and if MC use is interrupted, cannabis use disorder [CUD] symptoms, e.g., craving, may develop. The current sample reported an average of 2.6 h past since last MC use, meaning that patients typically used MC at multiple times during the day. Similar findings have been shown in other research with MC patients in Israel. A study with migraine patients licensed for MC treatment for a median period of 3 years found that patients who responded well to the MC treatment (i.e., reduction on migraine attacks after onset of MC use) used MC 5 times per day on average (range 2.5–7) whereas non-responders reported using 4.5 times daily (range 3–6). The difference between the groups was not significant (32). It is possible that frequent long-term use similar to that observed in the current and previous studies with CP patients leads to tolerance and CUD which in turn explains continued use despite lack of clear pain reduction association. Tolerance, CUD, as well as positive affect and quality of life warrant future research.

An alternative interpretation of the results is that MC products have analgesic effect, but that the current study analyses were unable to capture this possibility. Pain preceding MC use may be relatively high, and cannabis may immediately reduce and stabilize it until it quickly peaks, prompting further use. The models and ESM assessments were signal-contingent, designed to detect pain response to MC where pain fluctuated and gradually increased prompting MC use, but not to detect the alternative response scenario of low stable pain—peak in pain—MC use. It was felt that requesting self-initiated completion of ESM questionnaires immediately prior to MC use would have overloaded participants. This aspect should be included in future studies as it may be more sensitive to detect effects of MC on pain.

A significant negative time trend for intention to use cannabis, pain interference and intensity and fatigue was observed. This may relate to reactivity (e.g., systematic changes in experiences). Akin to mindfulness training, answering questions regarding pain and cannabis use may increase self-awareness, and influence participant's felt experience. Reactiveness has been found in daily diary studies of alcohol use (33). In one study reactiveness was found for alcohol use that tends to fluctuate across days, but not for marijuana use that tends to be more consistent (34). Yet, results similar to what can be expected from reactiveness have been found in other ESM studies of non-medical cannabis use (11, 35). ESM could theoretically be used as a self-monitoring tool for health promotion (36), a subject warranting future research.

Sex was not found to be a moderating factor vis-à-vis cannabis analgesic effect. The literature with human subjects is limited and contradictory, some reports showing greater analgesic effects in women (15, 30), others showing greater effects in men (16), warranting further study.

### Limitations

The micro-longitudinal design enabled to examine momentary experiences over time within-persons and with high external validity. Nevertheless, it only includes data from long-term MC users. The association of MC with pain reduction in novel users was not tested. The study lacks data on pain and other symptom levels immediately prior to MC use. Observational studies of MC use in naturalistic settings, capturing pain levels immediately prior to, and within hours after MC administration have found evidence of pain relief (30, 37, 38). The study also lacks data on symptoms of CUD. Positive affect was not assessed, given the necessity of keeping momentary assessments short. Increased positive affect after MC use has been found in a previous study (39), and should be tested in future studies of long-term MC use for CP along with MC analgesic effects in short- and long-term users.

Although we do not have a reason to expect that patients would change their reported last month opiate use during the study period, we did not collect daily data on prescription opiate use. This decision was made in order to keep the daily questionnaires as short as possible and prevent respondent fatigue. Thus, while we control for between-person last month prescription opiate use, daily within-person fluctuations in opiate prescription medication use was not controlled for.

The current research did not enable causality testing. Furthermore, although of reasonable size to detect within-person associations, we lack power to rigorously test between-person associations and cross-level interactions which highlight the need for replication of results reported here in larger samples. While the data on between-person differences in mode of administration was collected, people may use different MC strains and modes for different situations (40, 41), suggesting the need for more detailed, and momentary information about MC product concentration and administration characteristics to test within-person associations with momentary symptom relief.

## Conclusions

This study used ESM to examine real-world MC use for CP. Although preliminary, the results suggest that after long term use patients use MC in response to momentary pain experiences, consistent with MC treatment guidelines. However, it appears that patients do not actually achieve pain or other symptom relief. Limited pain relief may be due to development of tolerance to the analgesic effects of cannabis. MC administration may reap other benefits for patients, such as relief of CUD symptoms or improved sense of well-being, although clear evidence awaits future work. Daily diary studies may prompt reactiveness and thus improve symptoms related to pain and lower intention and actual use of MC. This needs to be tested directly in future studies.

## Data Availability Statement

The raw data supporting the conclusions of this article will be made available by the authors, without undue reservation.

## Ethics Statement

The studies involving human participants were reviewed and approved by the institutional review board (IRB) of the Faculty of Social Welfare & Health Sciences, University of Haifa (Certificate No. 304/19). The patients/participants provided their written informed consent to participate in this study.

## Author Contributions

SS conceptualized and designed the study as well as led the analysis and interpretation of data. SS led the drafting of the article and approved the final version. DR contributed to data collection, participated in the conceptualization and design of the study as well as the analysis and interpretation of data. SV participated in the analysis and interpretation of data. DM and TG participated in the conceptualization and design of the study as well as the analysis and interpretation of data and writing of the paper. All authors contributed to the article and approved the submitted version.

## Funding

This work was supported by the Evelyn Lipper Foundation (Grant No. 2027093). The grant was received by DM. The funding agencies were not involved in the design, analyses or interpretation of results.

## Conflict of Interest

The authors declare that the research was conducted in the absence of any commercial or financial relationships that could be construed as a potential conflict of interest.

## Publisher's Note

All claims expressed in this article are solely those of the authors and do not necessarily represent those of their affiliated organizations, or those of the publisher, the editors and the reviewers. Any product that may be evaluated in this article, or claim that may be made by its manufacturer, is not guaranteed or endorsed by the publisher.

## Abbreviations

CBD: Cannabidiol
CLBP: Chronic low back pain
CP: Chronic pain
CRPS: Complex regional pain syndrome
CUD: Cannabis use disorder
ESM: Experience sampling method
MC: Medical cannabis
NIS: New Israeli shekel
RCT: Randomized controlled trial
SD: Standard deviation
THC: Tetrahydrocannabinol
USD: U.S. dollar.
